# Content Quality of YouTube Videos About Metastatic Breast Cancer in Young Women: Systematic Assessment

**DOI:** 10.2196/45101

**Published:** 2023-11-14

**Authors:** Nina Morena, Yitzchok Ahisar, Xena Wang, Diana Nguyen, Carrie A Rentschler, Ari N Meguerditchian

**Affiliations:** 1 Art History and Communication Studies Faculty of Arts McGill University Montreal, QC Canada; 2 General Surgery Faculty of Medicine University of British Columbia Vancouver, BC Canada; 3 Faculty of Medicine McGill University Montreal, QC Canada; 4 Research Institute of the McGill University Health Centre Montreal, QC Canada; 5 St Mary’s Research Centre Montreal, QC Canada; 6 Gerald Bronfman Department of Oncology Faculty of Medicine McGill University Montreal, QC Canada; 7 Department of Surgery Faculty of Medicine McGill University Montreal, QC Canada

**Keywords:** social media, YouTube, metastatic breast cancer, breast cancer, patient education, health education, patient literacy, media literacy, health literacy, consumer health information, assessment tool, treatment, false information, women, videos, web-based

## Abstract

**Background:**

Young women with metastatic breast cancer (MBC) are part of a digitally connected generation yet are underserved in terms of information needs. YouTube is widely used to find and identify health information. The accessibility of health-related content on social media together with the rare and marginalized experiences of young women with MBC and the digital media practices of younger generations imply a considerable likelihood that young women with MBC will seek information and community on the internet.

**Objective:**

This study aims to assess the content quality of MBC YouTube videos and to identify themes in the experiences of young women with MBC based on YouTube videos.

**Methods:**

A systematic assessment of MBC YouTube videos using the search term “metastatic breast cancer young” was conducted in August 2021. The search was performed in an incognito browser and with no associated YouTube or Google account. Search results were placed in order from most to least views. Title, date uploaded, length, poster identity, number of likes, and number of comments were collected. Understandability and actionability were assessed using the Patient Education Materials Assessment Tool (PEMAT); information reliability and quality were assessed with DISCERN. Themes, sponsorships, and health care professionals’ and patients’ narratives were reported.

**Results:**

A total of 101 videos were identified. Of these, 78.2% (n=79) included sponsorships. The mean PEMAT scores were 78.8% (SD 15.3%) and 43.1% (SD 45.2%) for understandability and actionability, respectively. The mean DISCERN score was 2.44 (SD 0.7) out of 5. Identified themes included treatment (n=67, 66.3%), family relationship (n=46, 45.5%), and motherhood (n=38, 37.6%).

**Conclusions:**

YouTube videos about young women with MBC are highly understandable but demonstrate moderate rates of actionability, with low reliability and quality scores. Many have a commercial bias. While web-based materials have limitations, their potential to provide patient support is not fully developed. By acknowledging their patients’ media habits, health care professionals can further develop a trusting bond with their patients, provide a space for open and honest discussions of web-based materials, and avoid any potential instances of confusion caused by misleading, inaccurate, or false web-based materials.

## Introduction

Breast cancer is uncommon among young women, a population that is more likely to be diagnosed with more advanced and aggressive cancer than postmenopausal women [1]. Young women diagnosed with metastatic breast cancer (MBC) under the age of 40 years are marginalized from more common presentations of breast cancer (ie, early stage, postmenopausal, or non-metastatic), and therefore bear informational vulnerability. This refers to one’s risk of encountering and being affected by information that is false, inaccurate, or taken out of context, which can be exacerbated by low media literacy levels. In a context where their diagnoses and disease experiences are rare and understudied, young women with MBC often turn to social media and web-based forums to find MBC-related information and community. Young women with MBC actively seek information about their diagnoses and turn to scientific research that is then discussed in web-based settings [2-5].

The accessibility of health-related content on social media together with the rare and marginalized experiences of young women with MBC and the digital media practices of younger generations imply a considerable likelihood that young women with MBC will seek information and community in web-based contexts, including forums and social media platforms. These factors pose a risk that young women with MBC will encounter health information that is incorrect, misleading, false, or removed from the appropriate context. Health information on social media is largely unregulated; its impact on patients’ understanding is difficult to measure and is largely dependent on one’s own literacy skills. YouTube is a video sharing company and social media platform that is widely used to find and identify health information [6].

The purpose of this study is to assess the content quality of YouTube videos about and by young women with MBC and to identify common themes in MBC experiences based on video content. Identifying common themes contributes to knowledge of the emotional, social, and financial effects imposed on young women with MBC, which can help to better define priorities in patient-centered research. The outcomes of this study include knowledge of the content quality of YouTube videos, including their strengths and weaknesses, and further understanding of the experiences of young women with MBC, which may provide indications as to their reasons for seeking information and community in web-based spaces. Situated within a broader framework of the impact of social media on health care decisions, this study offers a perspective on the potential of social media regarding information circulation in cancer care among members of a vulnerable population.

## Methods

### Ethical Considerations

Ethics approval was not required as research involving publicly available data is exempt from McGill University’s Research Ethics Board review [7].

### Data Collection

A systematic assessment of YouTube videos with the search term “metastatic breast cancer young” was conducted on August 3, 2021. The search was performed in an incognito browser with no associated YouTube or Google account. Search results were placed in order from most to least views. The title, date uploaded, length, poster identity, number of likes, and number of comments were collected in a spreadsheet.

Several video characteristics were recorded. Videos included in playlists were recorded. Playlists are a collection of audio and video files created by users; they tend to be grouped together by theme and are intended to be watched in sequential order. Videos were also classified as information-based or experience-based. Information-based videos were driven by information and knowledge transfer, such as instances of health care professionals explaining a particular element of care or delivering a research presentation. Experience-based videos were driven by people’s experiences and stories, such as interviews with survivors or patients. The two were not mutually exclusive; a video could be described as both information- and experience-based. Reviewers took note of the presence of information and experience in order to account for the types of perspectives being offered in each video. In addition, reviewers noted whether videos included personal narrative, were educational, or offered advice. Personal narrative was defined as the presence of details about one’s lived experience; this differed from an experience-based video in that a video might be information-based but include mention of someone’s personal experience (eg, a physician giving a research talk who tells a short, personal anecdote). If a video was educational, this means it included the presence of knowledge transfer; this differed from a video being information-based in that a video might be experience-based but include some element of knowledge transfer (eg, a panel led by survivors or patients who discuss their experience of illness but also discuss how their treatment works or what their diagnoses mean). For a video to offer advice, it had to suggest that the viewer take some sort of action. Moreover, reviewers noted whether a video was part of a news media broadcast.

### Assessment Using the Patient Education Materials Assessment Tool and DISCERN

A review of selected videos was performed by a communication studies researcher and two health care professionals. All reviewers were trained to use the scoring instruments by the same person, including theme identification. Reviewers scored a small sample of videos collaboratively to establish reliability. Any major disagreements were resolved through consensus.

Content quality of the YouTube videos was assessed using the Patient Education Materials Assessment Tool (PEMAT) and DISCERN instruments. Understandability and actionability were scored using the PEMAT for audio-visual materials [8,9]. The PEMAT instrument offers a score for understandability and a score for actionability. Actionability refers to whether the material describes an action the viewer can take and whether it describes and explains steps toward taking that action. Each item in the PEMAT instrument was assessed and given a score of 0 (disagree) or 1 (agree). Scores were then added up and averaged to determine overall understandability and actionability. Information reliability and quality were assessed with DISCERN [10,11]. When assessing material using DISCERN, reviewers assigned a score that ranged from 1 to 5, where 1 was low and 5 was high. In both instruments, a higher score indicated higher quality levels. The PEMAT and DISCERN were only applied to the YouTube videos in the data set and not to the surrounding materials, such as titles, captions, or comments.

### Themes, Narratives, and Sponsorships

For each video, the themes addressed, presence of sponsorships, and health care professionals’ and patients’ narratives were also reported. Reviewers began identifying themes deductively with a predetermined list of themes of particular interest, defined in Table 1, and those that were most likely to appear in videos about the experiences of young women with MBC. The themes in Table 1 were collectively agreed upon at the research design stage. Subthemes were identified inductively based on notes taken during viewing that diverted from or were more specific than the main themes listed in Table 1. Sponsorships were identified as overt or covert. Overt sponsorships refer to explicit verbal mentions of an institution or company. Covert sponsorships refer to nonverbal instances of promotion, such as a banner, logo, or website URL appearing in the video. The presence of sponsorships was assessed in the video itself, and not in the surrounding description or caption.

**Table 1:** Theme definitions.

Theme	Definition
Treatment	Refers to a wide array of topics, ranging from treatment choices and side effects to the experience of receiving treatment
Family relationship	Refers to family-based experiences, such as how one may disclose their diagnosis to their family and how a diagnosis shifts the family dynamic
Motherhood	Refers to the specific relationship between mother and child, how to disclose to one’s children, as well as wanting to be a mother
Terminal status	Refers to the fact that one’s cancer has metastasized and may become their cause of death
Path to diagnosis	Refers to the story or experience of being diagnosed with breast cancer, such as discovering a breast lump
Spousal relationship	Refers to the patient’s relationship with their spouse, including stress on the spouse who takes on a caregiving role

## Results

### Data Collection

In total, 101 videos were identified (Table 2). Of these, 61 (60.4%) videos were information-based and 59 (58.4%) were experience-based. The average video length was 14.9 (SD 22.5) minutes. Most videos (n=96, 95%) were created and posted by an organization. The majority of videos were uploaded by nonprofit groups and breast cancer advocacy organizations, such as Rethink Breast Cancer. The group that uploaded the most videos was Living Beyond Breast Cancer (n=16, 15.8% of total videos; Table 3). Of the 6 YouTube channels in Table 3, 5 corresponded to organizations located in the United States; Rethink Breast Cancer was the only Canadian organization with significant channel frequency. The use of hashtags was not common; only 5.9% (n=6) of videos incorporated their use. Playlists were also uncommon; 23 (22.7%) videos were listed in a playlist, while 78 (77.2%) videos were not. Many videos included personal narrative (n=67, 66.3%) and were educational (n=64, 63.3%). News media clips were not common, as only 7 (6.7%) videos consisted of news media.

**Table 2:** Descriptive findings of YouTube videos (N=101).

Variable	Value
Identifiable corporate sponsorships, n (%)	79 (78.2)
Patient narrative, n (%)	64 (63.3)
Health care professional narrative, n (%)	58 (57.4)
Video length (minutes), mean (SD)	14.9 (22.5)
Number of viewer comments, mean (SD)	15.5 (69.0)
Number of viewer likes, mean (SD)	92.6 (415.0)

**Table 3:** YouTube channels by frequency.

Channel	Frequency (N=101), n (%)
Living Beyond Breast Cancer	16 (15.8)
NCCN^a^	15 (14.8)
Vital Options International	12 (11.9)
Young Survival Coalition	11 (10.9)
Rethink Breast Cancer	7 (6.9)
Dana-Farber Cancer Institute	3 (3)
Other^b^	37 (36.6)

^a^YouTube channel of the National Comprehensive Cancer Network.

^b^Channels present <3 times, including Nalie, Good Morning America, Refinery29, Gajendra Singh, MD, TODAY, Geisinger, Metavivor Online, TEDx Talks, Today’s Parent, Novartis, Susan G. Komen, Momjo, Cleveland Clinic, European Society for Medical Oncology, SELF, Rachel Leigh, Whitehead Institute for Biomedical Research, KGUN9, dailyRx, Cancer Support Community, Tigerlily Foundation, Icon Cancer Centre, Metastatic Breast Cancer Alliance, Gulf States Young Breast Cancer Survivor Network, First Coast News, vcbf1991, WTKR News 3, Breaking News, Ascension Seton, CancerFightClub, UCLA Health, Phoenix Children’s Hospital, and DNA Today.

### Assessment Using PEMAT and DISCERN

The mean PEMAT audio-visual scores were 78.8% (SD 15.3%) and 43.1% (SD 45.2%) for understandability and actionability, respectively (Table 4). Overall, videos had moderate reliability and quality levels, and the mean DISCERN score was 2.44 (SD 0.7) out of 5.

**Table 4:** Distribution of Patient Education Materials Assessment Tool (PEMAT) and DISCERN scores.

Tool			Mean (SD)		Range		Median (IQR)		Mode
**PEMAT understandability**									
	Total points	7.6 (1.9)		3-12		7.0 (5.0-9.5)		7.0	
	Total possible points	9.9 (1.2)		5-12		10.0 (7.5-11.0)		10.0	
	Score (%)	78.8 (15.3)		30-100		77.8 (53.9-88.9)		70	
**PEMAT actionability**									
	Total points	1.4 (1.4)		0-4		1.0 (0.5-2.5)		0.0	
	Total possible points	3.1 (0.3)		3-4		3.0 (0-0.5)		3.0	
	Score (%)	43.1 (45.2)		0-100		33.3 (16.7-66.7)		0	
**DISCERN**									
	Total points	39.0 (11.1)		18-68		39.0 (28.5-53.5)		38.0	
	Average out of 5	2.4 (0.7)		1.1-4.3		2.4 (1.8-3.4)		1.8	

### Themes, Narratives, and Sponsorships

Commonly identified themes included treatment (67/101, 66.3%), family relationship (46/101, 45.5%), motherhood (38/101, 37.6%), terminal status (32/101, 31.6%), the path to diagnosis (29/101, 28.7%), and spousal relationship (25/101, 24.7%; Table 5). Subthemes included feelings of stress, anxiety, depression, and other mental health issues; racial disparities in breast cancer; making arrangements for end of life; fear of progression; explaining what it means to be “stage IV”; the “pink” movement in breast cancer; as well as participation in clinical trials and research.

**Table 5:** Thematic findings.

Theme	Prevalence (N=101), n (%)
Treatment	67 (63.3)
Family relationship	46 (45.5)
Motherhood	38 (37.6)
Terminal status	32 (31.6)
Path to diagnosis	29 (28.7)
Spousal relationship	25 (24.7)

Patient narratives were shared in 63.3% (64/101) and health care professional narratives in 57.4% (58/101) of videos. Of the videos that included patient narratives, 54.7% (35/64) provided a diagnosis timeline, 7.8% (5/64) were recently diagnosed (roughly within a year of the video being posted to YouTube), and 29.7% (19/64) had a diagnosis date over a year prior to the video being posted to YouTube. Advocate narratives were present in 28.7% (29/101) of videos. Scientist narratives were present in 7.9% (8/101) of videos. Scientists were distinguished from health care professionals as individuals who were identifiable (by their own introduction) as researchers who are not clinicians and do not provide care to patients directly. Overall, 78.2% (79/101) of videos were sponsored. Of the 79 sponsored videos, 22 (27.8%) were covert sponsorships and 57 (72.2%) were overt sponsorships.

## Discussion

### Principal Findings

Young women with MBC represent an uncommon presentation of disease among a highly digitally connected generation. We showed that YouTube videos about MBC were very understandable but demonstrated low to moderate rates of actionability, with low reliability and quality scores. Videos were also often sponsored. Our findings hold implications for the role and possible benefits of social media in cancer care. Our study contributes to a range of existing methods to assess information quality [12]. We combined the use of standardized instruments with a qualitative thematic approach in order to gain an understanding of patient experiences and concerns relative to video content quality. Given the often-unregulated nature of YouTube content and of web-based information more broadly, evaluating YouTube videos with validated instruments provided an opportunity to measure the strengths and weaknesses of YouTube videos.

The high PEMAT understandability score, which implies that videos are clear and accessible in language, is a reminder that YouTube videos are popular because they are easy to watch and understand. Patients are generally satisfied with oncology services, though research suggests that improvement in the explanation of long-term side effects, treatment options, and support with psychological, emotional, and physical elements of cancer would be beneficial [13]. While web-based materials do not represent a direct contrast to visits with one’s oncologist, there are important distinctions between the two forms of information delivery. Indeed, an internet search and a conversation with one’s oncologist represent vastly different information environments; the former is completely driven by the patient, is voluntary, and is readily accessible at any time, whereas the latter is scheduled, limited by time constraints, and occurs at the discretion of the oncologist. In this way, internet searches may represent an addition to the information that is provided by the oncologist and care team and do not necessarily imply that the patient is choosing to dismiss information provided by their medical care team. Internet searches may also be a way for patients to navigate complex medical information. A literacy assessment of the National Comprehensive Cancer Network (NCCN) guidelines on the management of the most common cancer diagnoses revealed that, while scoring high on the PEMAT scale, the guidelines have a reading level higher than what is considered suitable for the general adult population of the United States [14]. Therefore, internet searches do not only indicate a need for more information but also represent an opportunity for alternative or additional understandable explanations. Heavy viewership of YouTube videos might be a signal that health care professionals need to communicate more clearly, but not in terms of providing accurate information; rather, they must ensure that they are conveying information to patients in an understandable and comprehensive way. Therefore, in cases where there is a communication barrier between patients and physicians, YouTube’s accessibility and clarity may act as a helpful complement to what the patient learns during their appointment.

The low levels of quality and reliability found in the videos analyzed in this study are characteristic of the overall troubling lack of regulation of web-based content and are consistent with other studies of YouTube video content quality. YouTube videos about breast cancer [15], prostate cancer [16,17], idiopathic pulmonary fibrosis [18], cleft lip and palate [19], hysterectomy [20], and neurotoxins [21] and educational videos about plastic surgery [22,23] are low in information quality. YouTube information quality is considered promising regarding food poisoning [24] and fair for orthodontic smile design [25]. YouTube videos about cosmetic surgery were shown to have high levels of bias and low levels of quality when measured with DISCERN [26]. Yuksel and colleagues [27] similarly demonstrated that YouTube videos about pregnancy and COVID-19 have many views but are low in quality and trustworthiness. Therefore, while YouTube content about health conditions is abundant, viewers should continue to be wary of its information quality. Although content moderation is part of every platform’s function, it cannot account for content that is potentially misleading but that does not violate any community guidelines [28]. Information considered to be “fake news” encapsulates a wide variety of information that exceeds information that is simply false and includes misreporting and persuasive information [29]. Content moderation often occurs after a post is already shared, and the processes and justifications behind it tend to be kept secret by platforms [30]. Therefore, the lack of oversight on health information in web-based spaces is a reality that patients and health care professionals alike must contend with. In addition, many videos have a potential commercial bias. Heavy sponsorship and corporate presence are common on YouTube, though they are also common in public messaging about breast cancer. Sponsorships may not always be easily discernible or recognizable by the average viewer, a phenomenon that merits further attention and concern.

Despite the existing low levels of quality among YouTube videos, the platform holds wide potential for communicating public health information to a large audience, as we have recently witnessed with COVID-19 mitigation [31]. Low levels of web-based information quality are going to persist; therefore, health care professionals should consider providing tools to enhance patient autonomy in assessing the web-based information they consume, especially considering research that demonstrates low levels of electronic and internet health literacy among cancer survivors [32]. Rather than strictly discouraging patients from searching the internet, patients should be made aware of responsible ways of using internet sources [33]. For example, learning how to recognize sponsorships as well as the motivations behind forms of web-based content can help to alleviate the effects of a lack of content regulation and decrease informational vulnerability. Similar to the prompts of the PEMAT and DISCERN instruments, questioning the purpose behind a video and how the information in the video is presented are habits that can contribute to higher levels of media literacy [34]. Media literacy education may not originate from the oncologist, however; as Tran and colleagues [14] explain, while clinicians are always important sources of information, there is little they can do to “directly help improve patients’ literacy skills.” Rather, libraries and educational institutions provide guides to navigating web-based content and identifying potential misinformation. For example, on its website, the Toronto Public Library offers a guide titled “How to Spot Fake News,” which also links to books, videos, and other research guides on misinformation [35].

The themes most commonly identified in our study are predominantly experiential, which may suggest that many interpersonal and relationship-based concerns—reflected in our findings as being prioritized by patients—are not being sufficiently addressed by health care professionals in structured clinical encounters, signaling an unmet need to connect to a patient community. Engaging on the internet, therefore, may act as evidence of diverging information priorities between the patient and the physician. For instance, Tran and colleagues [14] cite a survey conducted by the NCCN that demonstrated that the information in the NCCN guidelines, which are comprised mainly of treatment details, did not align with what patients were looking for [36].

That many of the identified themes are experiential also suggests that content about the lived experience of young women with MBC is both successful and desired by patients, in addition to information about the disease itself. Personal stories on social media are very common, reflecting the importance of finding community on the internet [37]. As Ginter [38] shows, young age combined with late-stage diagnosis represents specific challenges for young women with MBC who face difficulties with short- or long-term decision-making. Young women with MBC struggle with anxiety [39], are susceptible to posttraumatic stress, and need social support [40]. Social media participation has the potential to assist in alleviating patient anxiety; indeed, Attai and colleagues [41] demonstrated that breast cancer patients’ “perceived knowledge increases and their anxiety decreases by participation in a Twitter social media support group.” Beyond MBC, the lived experience of patients with metastatic lung cancer is understudied [42]. A study by Petrillo and colleagues [43] on the experience and supportive care needs of people with metastatic lung cancer concluded that patients with metastatic nonsmall cell lung cancer who receive targeted therapy, as well as their caregivers, “experience distress related to living with uncertainty and desire more coping support, connection with peers, information, and healthy lifestyle guidance.” Their findings, which indicate the need to develop tailored support services, highlight the ways in which the experience of living with metastatic disease is understudied, unique, and requiring of specific forms of support. Moreover, assessing the themes and topics discussed in web-based spaces may prove useful for policy development or improvements to patient care [4].

### Limitations

Limitations of this study include restrictions based on language, country of origin, and quality assessment. This study included only English-speaking videos, many of which originated in a US context, and therefore reflects specific social and geographical points of view. In addition, the use of the PEMAT and DISCERN instruments limited our assessment to only the videos themselves. We recognize that the surrounding content, such as captions and comments, may potentially contain rich information. This content represents an opportunity for future research, as it documents viewer reactions and may provide insight into how the viewers choose to process information that may affect their health.

### Practical Implications

Our findings hold important implications for communication practices in oncology. Per policy recommendations in the realm of cancer literacy, health care professionals (oncologists in particular), where feasible, should be sensitive and receptive to the knowledge patients have gained through their internet searches and social media participation and improve their communication skills in this area [33]. By acknowledging their patients’ media habits, health care professionals can potentially further develop a trusting bond with their patients by including them in setting the priorities for each appointment, providing a space for open and honest discussions of web-based materials, and avoiding any potential instances of confusion caused by misleading, inaccurate, or false web-based materials. These communication practices can help patients to be better equipped in their internet searches and social media participation and improve their ability to discern sponsorships and commercial messaging. Moreover, research indicates that trust between patient and physician is reciprocal and that communication quality has a significant influence on building that trust [44]. Furthermore, in attending to patients’ media habits and practices, health care professionals can have the opportunity to stay informed on what is currently trending or popular regarding cancer in web-based spaces.

### Conclusion

Social media use and participation in internet searches are widespread habits that are well-established and sure to remain an important part of the experience of disease, in particular among younger populations. While web-based materials have limitations, including high rates of sponsorship bias and low levels of information quality, their potential to provide patient support is not fully developed. More research is needed to evaluate the impact of YouTube videos on patient decisions and possible interventions provided by health care institutions. Future research may include patients’ perspectives on these findings and on YouTube as a platform for information and community-seeking.

## Abbreviations

MBC: metastatic breast cancer
NCCN: National Comprehensive Cancer Network
PEMAT: Patient Education Materials Assessment Tool

## Data Availability

The data sets generated and analyzed during this study are available from the corresponding author upon reasonable request.
